# Plasma amino acids after human milk fortification and associations with growth in preterm infants

**DOI:** 10.1038/s41390-025-04126-6

**Published:** 2025-05-18

**Authors:** Martin Bo Rasmussen, Kristine Holgersen, Tik Muk, Azra Leto, Allan Stensballe, Gerrit van Hall, Lise Aunsholt, Susanne Soendergaard Kappel, Gitte Zachariassen, Per Torp Sangild

**Affiliations:** 1 Comparative Pediatrics, Section for Biomedicine, Department of Veterinary and Animal Sciences, University of Copenhagen, Frederiksberg, Denmark; 2 Hans Christian Andersen Children’s Hospital, Odense University Hospital, Odense, Denmark; 3 Department of Health Science and Technology, Aalborg University, Aalborg, Denmark; 4 Sino-Danish Center, Beijing, China; 5 Clinical Cancer Research Center, Aalborg University Hospital, Aalborg, Denmark; 6 Clinical Integrative Fluxomics core, Clinical Biochemistry, Rigshospitalet Copenhagen, Denmark; 7 Department of Biomedical Sciences, University of Copenhagen, Copenhagen, Denmark; 8 Department of Neonatology, Rigshospitalet Copenhagen, Denmark; 9 Department of Clinical Medicine, University of Copenhagen, Copenhagen, Denmark; 10 Department of Clinical Research, University of Southern Denmark, Odense, Denmark; 11 Open Patient Explorative Network, Odense, Denmark; 12 Centre for Science and Faith, Faculty of Theology, University of Copenhagen, Copenhagen, Denmark

## Abstract

**Background:**

It is unknown how plasma amino acid (AA) concentrations vary with fortification type, growth and insulin-like growth factor 1 (IGF-1) concentrations in the first weeks of life in very preterm infants (VPIs).

**Methods:**

Human milk for VPIs (*n* = 225) was fortified with bovine colostrum (BC, intact proteins, high bioactivity) or conventional fortifier (CF, hydrolysed bovine whey proteins). Plasma was sampled at fortification start (T0, ~1 week of age) and after one (T1) and two (T2) weeks. Changes in Z-scores for weight, length and head circumference (HC) were calculated from T0 to 35 weeks postmenstrual age.

**Results:**

Compared with CF, BC fortification increased 12 AAs (~10–40%, *p* < 0.05) and reduced Lys concentrations (10–16%, *p* < 0.05). Analysed across groups, T0-T2 AA increments associated positively with HC growth (12 AAs) and IGF-1 concentrations (5 AAs), and inversely with gestational age (13 AAs) and weight (8 AAs) at birth. The plasma protein profile (proteome) was unaffected by fortification.

**Conclusions:**

BC fortification increased the plasma concentrations of many AAs. Fortification-induced AA increments associated positively with HC growth and IGF-1 concentrations, and were affected by immaturity and birth weight. Still, plasma AA variability within physiological levels appears to have limited implications for clinical outcomes during the early life of VPIs.

**Impact:**

- It is unknown how human milk fortification affects plasma amino acid concentrations, in turn influencing growth patterns in very preterm infants.
- We show that a fortifier based on bovine colostrum induces higher amino acid concentrations than a conventional fortifier.
- Fortification-induced increments in amino acid concentrations associated with gestational age, birth weight and head growth, but with small effect sizes and limited relation to body weight or length growth.
- Plasma amino acid concentrations are influenced by fortification of human milk in early life, but have limited practical application as predictors of body growth and health in individual very preterm infants.

## Introduction

Human milk (mother´s own milk, MOM, or donor human milk, DHM) fed to hospitalized very preterm infants (VPIs, <32 weeks of gestational age, GA) is typically nutrient-fortified with protein, energy and minerals. This is to improve their growth and development, as indicated by small, but significant, increases in body weight, length and head circumference (HC),^1,2^ thereby avoiding the postnatal growth deficits often observed in VPIs.^3^ The plasma levels of specific amino acids (AAs) are the combined result of absorption from the gut and de novo synthesis (non-essential AAs), together with metabolism, breakdown and excretion. While essential AAs (EAAs: Lys, Met, Thr, Leu, Ile, Val, Phe, His, Trp) are exclusively derived from the diet, others are semi-essential (i.e., essential only under certain conditions: Arg, Gly, Pro, Tyr, Cys, Gln).^4^ In contrast, individual proteins in plasma are almost exclusively of endogenous, mainly hepatic, origin and therefore not directly related to protein supply from the milk diet. Previous studies on dietary protein and AA supplementation in preterm infants have mostly assessed the effects of crude protein supply or total AA intake on growth. However, specific changes in systemic AA concentrations or AA groups, like branched-chain AAs (BCAAs: Leu, Ile, Val), may also be key drivers for postnatal growth. Besides functioning as building blocks for tissue protein, AAs stimulate the growth indirectly via AA-specific effects on protein synthetic pathways^5–9^ or paracrine/endocrine growth promotors, such as insulin and insulin-like growth factor 1 (IGF-1).^10–12^ On the other hand, excessive supply of enteral and/or parenteral nutrition (PN) may predispose to late-onset sepsis (LOS)^13,14^ and there is concern that excessive intake of protein or AAs may lead to toxic levels of certain AAs, like in hypertyrosinemia.^15^ Thus, it is critical to provide sufficient AAs for protein synthesis, bioactivity and energy, and at the same time, avoid excessive supply.^16^ An improved understanding of fortification-induced AA responses may guide fortification in preterm infants towards healthy growth and development.

Bovine colostrum (BC) has been suggested as an alternative to conventional fortifier (CF) due to its high contents of milk protein and bioactive components, like immunoglobulins and growth factors.^17–19^ BC used as a fortifier to human milk markedly improved growth, gut and immune development in the first weeks of life in preterm pigs, used as a model for preterm infants.^20,21^ However, when VPIs were supplemented with BC from a few days after birth, the clinical benefits to growth, gut function and morbidities were minimal or absent, especially when significant amounts of MOM were available.^22^ In a recent randomized clinical trial (FortiColos^23^), VPIs fed MOM or DHM fortified with BC showed a different plasma AA profile after two weeks of fortification than infants receiving CF. The fortifier amount and composition could play a role in elevated AA concentrations (e.g., more casein and immunoglobulins in BC versus CF^23,24^), as well as the differences in AA digestibility (e.g., protein pre-hydrolysis only for CF). Protein and AA supply may also affect endogenous protein and AA metabolism in specific organs, including the gut and liver.^25,26^

On this background, we assessed the effects of BC fortification on circulating AAs and proteins. As an explorative part of the study, we investigated how fortification-induced AA changes during the first two weeks of fortification were associated with changes in anthropometric measures, immaturity (GA and weight at birth), key morbidities (bronchopulmunary dysplasia, BPD, retinopathy of prematurity, ROP, LOS) and IGF-1 plasma concentrations.

## Methods

### Study population and ethics

The study was a secondary analysis of data and samples from the FortiColos trial (ClinicalTrials.gov: NCT03537365), an open-label randomized controlled multicentre pilot trial, comparing fortification with BC (ColoDan powder, Biofiber-Damino, Gesten, Denmark) and CF (PreNAN, FM85, Nestlé) in VPIs (GA: 26 + 0 to 30 + 6 weeks).^23,27^ Inclusion criteria were enteral intake >100 mL/kg/d at the start of fortification, clinical condition allowing for fortification, no major congenital anomalies or gastrointestinal surgery and no previous formula intake. The goal of the trial was to evaluate the feasibility and safety of BC fortification. The sterility of the BC product was achieved by gentle spray-drying, low-temperature pasteurization (63 °C, 30 min) and gamma irradiation. The procedures have been described to preserve the bioactivity of the product relative to standard pasteurization.^24,28,29^ Pre-prandial blood samples were collected at three time points, at the start of fortification (T0, median postnatal age 7 days, *n* = 210), after one week (T1, 7 ± 3 days, median postnatal age 16 days, *n* = 216) and two weeks (T2, 14 ± 3 days, median postnatal age 22 days, *n* = 199) of fortification. The ethical committee of the Region of Southern Denmark (S-20130010, S-20170095) and the Danish Data Protection Agency (17/33672) approved the study. Written parental consent was obtained for all participants.

### Infant feeding and milk fortification

A detailed description of the study protocol and fortification process has been published previously.^23,27^ Local clinical nutritional guidelines, as adapted from ESPGHAN,^30^ were followed, starting MOM feeding as soon as possible after birth, supplemented with DHM when needed. Fortification of MOM or DHM started at median postnatal day 8-9 when enteral intakes reached 100–140 mL/kg/d and blood urea nitrogen (BUN) was <5 mmol/L. This was the same as the average age at which infants, who received PN, stopped receiving it.^23^ Infants received either BC or CF fortifier every 2–3 hours. Feeding volumes and amounts of fortification aimed to reach growth adhering to existing guidelines at each participation neonatal unit (i.e., not fixed by the protocol). Contents of AAs in the two fortification products are shown in Table 1. The intervention continued until postmenstrual age (PMA) 34 + 6 weeks.

**Table 1:** Composition of macronutrients and amino acids in conventional fortifier (CF) and bovine colostrum (BC) powder.

Nutrients	CF content per 4.0 g	BC content per 2.8 g	BC/CF ratio
Energy, kcal	17.0	13.0	0.77
*Macronutrients, g*			
Carbohydrates	1.30	0.48	0.37
Lipid, g	0.70	0.64	0.91
Protein, g	1.4	1.4	1.00
- of which immunoglobulin G, g	-	0.62	-
- of which casein, g	-	0.44	-
*Amino acids, mg*			
Ala	79	52	0.66
Arg	34	53	1.56
Asp	169	109	0.65
Glu	225	208	0.92
Gly	22	33	1.50
His	28	33	1.18
Ile	79	60	0.76
Leu	186	123	0.66
Lys	170	106	0.62
Met	34	28	0.82
Phe	55	62	1.13
Pro	64	108	1.69
Ser	65	99	1.52
Thr	76	78	1.03
Tyr	46	66	1.44
Val	78	99	1.27

Macronutrient concentrations were provided by the manufacturers and amino acid concentrations were analysed separately after product hydrolysis. Values are provided for the maximal amount of fortifier protein (+1.4 g) added to 100 mL of human milk (equivalent to 4.0 g CF and 2.8 g BC).

The time points selected after the start of fortification (T1 and T2) were chosen to monitor the changes in AA and IGF-1 concentrations in the initial fortification period. The safety of BC fortification was assessed by comparing plasma AA concentrations after BC and CF fortification with the range observed in another study in preterm infants with clinical conditions similar to the present study. Infants in the chosen reference study had a mean GA at birth of 30.7 weeks, a mean postnatal age of 16 days and showed positive energy balance and normal growth rates when human milk was fortified with a fortifier identical to our control fortifier.^16^

### Clinical data collection and calculations

Clinical data on anthropometry (weekly weight in gram, length in cm, HC in cm) from birth to end of intervention, and information on BPD (oxygen supplementation or respiratory support at 36 weeks PMA) and ROP (diagnosed by attending ophthalmologists) were registered in an OPEN REDCap database. Cases of LOS after start of fortification were defined as antibiotic use for more than five days. As stated in the original clinical paper,^23^ the number of infants diagnosed with morbidities related to prematurity (e.g., BPD, ROP, LOS, necrotizing enterocolitis, intraventricular haemorrhage) did not differ between the BC and CF group. Information about the amount of fortification, enteral volume and type of milk (MOM, DHM) was retrieved via nurse-registered case reports. Enteral milk and protein intakes were calculated, assuming protein contents of 1.7 g/100 mL in MOM, 0.9 g/100 mL in DHM and 1.3 g/100 mL when the provided milk was a mixture of MOM and DHM. The type of milk on any given day was set as the most frequent milk type fed on that day. Combining data from the two fortification groups, changes in AA concentrations from T0 to T2 and associations with growth parameters, immaturity, and morbidities were assessed in infants with plasma samples and AA measurement available at all three sampling time points (*n* = 182) to avoid possible bias of missing data for individual infants.

Anthropometry data were converted to Z-scores for weight, length and HC, using a Swedish continuous sex specific growth ref.^31^ Linear interpolation between each weekly anthropometry measurement was used to estimate weight, length, HC and their respective Z-scores for all days. To assess the effects of fortification-induced changes in AA concentrations on growth, we associated the AA changes between T0 and T2 with changes in Z-scores for weight, length and HC from the start of fortification (T0) to the end of fortification at 35 weeks PMA. Small for gestational age (SGA) was defined as birth weight Z-score ≤ -2 standard deviation (SD).

### Blood sampling and analyses of AAs and IGF-1

Blood samples (~500 µL) were collected in EDTA tubes, kept on ice and centrifuged (2500 x g, 4 °C, 10 min) to yield plasma. Plasma was frozen at −60° to −80 °C for later measurement of the 20 AA concentrations by reverse-phase high-performance liquid chromatography.^32^ Units of AAs are given in µmoI/L. Concentrations of IGF-1 in plasma were determined using an enzyme-linked immunosorbent assay (ELISA, E-20, Mediagnost, Reutlingen, Germany). Intra- and inter-assay variability was 5.8% and 6.2%, respectively, as declared by the manufacturer. IGF-1 values are given in ng/mL. Limitations on allowed blood sample volumes led to differences in the number of samples available for IGF-1 measurement.

### Relative-quantitative assessment of plasma proteins by mass-spectrometry proteomics

Plasma was diluted 1:10 fold with cold lysis buffer (1% Sodium dodecyl sulfate, 100 Mm ammonium bicarbonate, 10 mM tris(2-carboxyethyl) phosphine, 40 mM 2-Chloroacetamide, pH 8.5) and prepared with an automated universal solid-phase-enhanced sample preparation (uSP3) method for liquid chromatography tandem mass spectrometry (MS) analysis, as previously described.^33^ Proteins were digested with trypsin/lys-C mix (1 µg/100 µg protein) at 37 °C and peptides were dried down in a vacuum centrifuge and reconstituted in MS loading buffer (2% acetonitrile in MS-grade water). Total peptide concentration was determined (Pierce Quantitative Peptide Assays Kit, Thermo Scientific, MA) and 400 ng peptide loaded onto Ultra-Performance Liquid Chromatography (Dionex RSLC Proflow, Thermo Scientific, MA), coupled with a timsTOF Pro (Bruker, MA). Proteins were identified using Spectronaut’s directDIA+ (Biognosys, MA) using default settings against the UniProt reference database (*homo sapiens*) at 1% FDR. Proteomic data were sorted into effect protein groups (i.e., specific proteins found in at least 70% of the samples per group) and exposure protein groups (less than 30% of the samples in one group and above 70% of the samples in the other group). The MS proteomics data have been deposited to the ProteomeXchange Consortium via the PRIDE partner repository with the dataset identifier PXD053348.

### Statistical analyses

Statistical analyses were performed with software SAS (Version 9.4, SAS Inst., Cary, North Carolina) or R (version 4.3.3, R Foundation for Statistical Computing, Vienna, Austria). Basic clinical characteristic differences between groups were assessed using students t-test or Mann-Whitney U test for continuous data or Chi-square test or Fisher´s exact test for binomial data. When needed to obtain normality, data were log-transformed for statistical analyses. General linear models were used to detect differences in AA concentrations and proteome profiles between fortification groups at each time point, adjusting for SGA, GA group at birth (above or below 29 weeks), region of inclusion and postnatal age at sampling. Pearson’s Chi-squared test or Fisher´s exact test for binomial data were used to assess group differences in the number of infants having specific AA concentrations outside a chosen reference level. General linear models were used to compare plasma changes in AA concentrations from T0 to T2 across fortification groups adjusting for intervention, SGA, GA group at birth and postnatal age at T2. Plasma AA associations with growth, IGF-1 and morbidities were analysed by general linear models adjusted for intervention, SGA, GA group at birth and postnatal age at T2. *P* values < 0.05 were regarded as statistically significant.

The differences in proteome profiles between sampling time points across fortification groups were examined using a linear mixed-effect model, where infant identifier was included as random factor and intervention, SGA, GA group at birth, region of inclusion and postnatal age at sampling were included as fixed factors. For proteomics data, *p* values were further adjusted with *q* values by the Benjamini-Hochberg method, adjusting for a false discovery rate (FDR, α = 0.2) and to control for potential type I errors. A threshold of *q* ≤ 0.1 was set to identify differentially expressed proteins (DEPs) between fortification groups.

## Results

### Effects of BC-based fortification on plasma AA concentrations

**Fig. 1:**
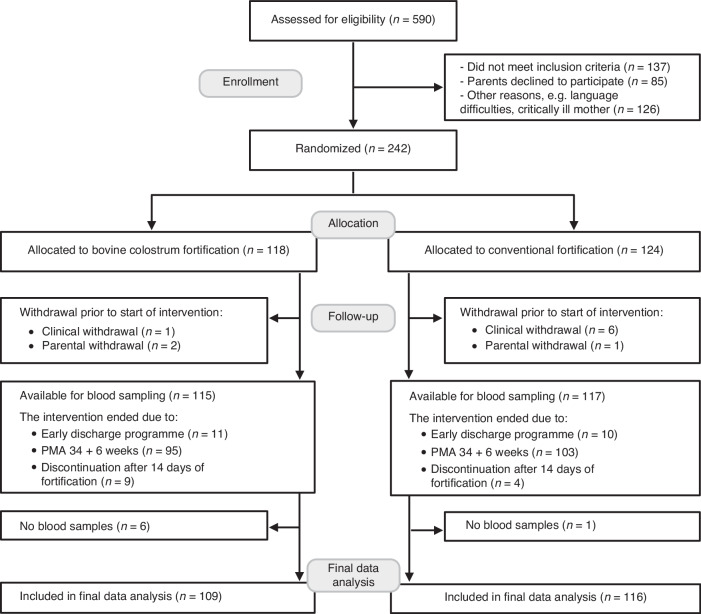
Participant flow chart. Created with BioRender.com.

Participant flow chart and clinical characteristics of the entire cohort included in the intervention study are shown in Fig. 1 and Table 2, respectively. The two fortification groups did not differ in their clinical characteristics, but differences in nutritional intake were observed. Compared with CF infants, BC infants received slightly higher enteral milk volumes (both *p* ≤ 0.01*)* and total enteral protein (*p* = 0.05–0.08*)* at T1 and T2. There was a tendency to a lower proportion of BC infants being fed primarily with MOM at T2 (*p* = 0.07, Table 2).

**Table 2:** Clinical and enteral nutrition characteristics in very preterm infants randomized to fortification with conventional fortifier (CF) or bovine colostrum (BC).

Clinical characteristics	*n*	CF	*n*	BC	*p*
*Perinatal variables*					
Median GA at birth, weeks + days (min-max)	116	28 + 5 (26 + 0–30 + 6)	109	28 + 6 (26 + 0–30 + 6)	0.60^a^
GA < 29 weeks at birth, *n* (%)	116	53 (45.7)	109	51 (46.8)	0.87 ^b^
Female, *n* (%)	116	52 (44.8)	109	44 (40.4)	0.50^b^
Multiple birth, *n* (%)	116	38 (32.8)	109	32 (29.4)	0.58^b^
Small for gestational age, *n* (%)	116	26 (22.4)	109	26 (23.9)	0.80^b^
Antenatal steroid, *n* (%)	116	109 (94.0)	109	106 (97.2)	0.34^c^
Amnionitis, *n* (%)	115	3 (2.6)	109	5 (4.6)	0.49^c^
Preeclampsia, *n* (%)	112	23 (20.5)	107	26 (24.3)	0.50^b^
Placental abruption, *n* (%)	114	7 (6.1)	107	11 (10.3)	0.26^b^
Preterm premature rupture of membranes, *n* (%)	116	25 (21.6)	109	24 (22.0)	0.93^b^
Caesarean section, *n* (%)	116	86 (74.1)	109	75 (68.8)	0.38^b^
Apgar < 7 after 5 minutes, *n* (%)	109	8 (7.3)	102	13 (11.9)	0.19^b^
*Anthropometry at birth*					
Mean weight at birth, g (SD)	116	1164 (324)	109	1172 (336)	0.87 ^d^
Mean weight Z-score (SD)	116	−1.1 (1.2)	109	−1.2 (1.2)	0.63 ^d^
Mean length Z-score (SD)	102	−1.4 (1.8)	97	−1.3 (2.0)	0.70 ^d^
Mean head circumference Z-score (SD)	104	−0.8 (1.0)	97	−0.7 (1.0)	0.57 ^d^
*Anthropometry at end of intervention (PMA 35 weeks)*					
Mean weight Z-score (SD)	116	−1.2 (1.1)	107	−1.4 (1.1)	0.12 ^d^
Mean length Z-score (SD)	107	−1.6 (1.6)	99	−1.9 (1.9)	0.61 ^d^
Mean head circumference Z-score (SD)	107	−0.6 (0.8)	97	−0.7 (1.1)	0.55 ^d^
*Enteral intake*					
Median enteral volume, ml/kg/day (min-max)					
T1	103	161 (109–251)	101	162 (135–224)	0.01^a^
T2	97	158 (0–209)	92	166 (99–235)	<0.01^a^
Mean protein provided from fortifier and human milk intake, g/kg/day (SD)					
T1	91	4.0 (0.8)	95	4.2 (0.7)	0.08 ^d^
T2	86	4.1 (0.7)	86	4.4 (0.8)	0.05 ^d^
Predominantly MOM feeding, n (%)					
T1	97	83 (85.6)	95	75 (78.9)	0.23^b^
T2	92	81 (88.0)	91	71 (78.0)	0.07^b^

*GA* gestational age, *PMA* postmenstrual age, *MOM* mother’s own milk, SD Standard deviation. ^a^Mann-whitney U test, ^b^Chi-squared test, ^c^Fishers exact test, ^d^Students t-test.

**Fig. 2:**
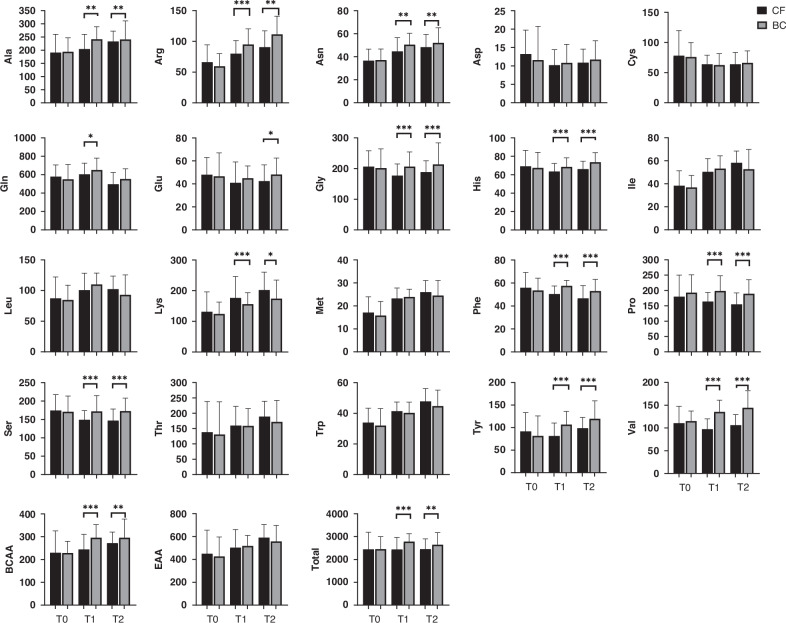
Fortification type (BC: bovine colostrum, CF: conventional fortifier) and plasma amino acids in very preterm infants. Plasma was sampled at the start of fortification (T0, CF: *n* = 110, BC: *n* = 100), and after one (T1, CF: *n* = 111, BC: *n* = 105) and two (T2, CF: *n* = 101, BC: *n* = 98) weeks of fortification and analysed by reversed-phase high-performance liquid chromatography. General linear models were used and adjusted for gestational age group at birth (above or below 29 weeks of gestation), small for gestational age, region of inclusion and postnatal age at sampling. Data is presented as median with interquartile range and all units are µmol/L. *p < 0.05, **p < 0.01, ***p < 0.001.

At T0, no differences in AA concentrations were observed between infants fortified with BC or CF (*n* = 100–110, Fig. 2). Infants fortified with BC had higher plasma concentrations of Ala, Arg, Asn, Gly, His, Phe, Pro, Ser, Tyr and Val, and reduced Lys concentrations, at both T1 and T2 compared with CF infants (*n* = 98–111, Fig. 2). Additionally, the concentrations of Gln and Glu were increased in BC infants at T1 and T2, respectively. This led to increases in the combined values for BCAAs and total AAs in the BC group. Asp, Cys, Ile, Leu, Met, Thr, Trp and total EAA concentrations were similar between the groups at both T1 and T2. Results remained similar after adjusting for baseline (T0) levels, except that Lys concentrations in BC infants were no longer lower at T2. After adjustment for total protein or enteral volume intake, Asn concentrations at T2 were no longer different between groups. Also, after adjustment for enteral volume intake, Ala concentrations at T2 were no longer different (data not shown).

When plasma AA concentrations at T2 were compared to those in the chosen reference group of infants,^16^ we found that BC fortification led to more infants having concentrations above the 95th percentile for Arg, Ser, Tyr and Val, relative to CF (all *p* < 0.05, Supplementary Table S1). One infant in the BC group, born at GA < 29 weeks and diagnosed with ROP (but no other morbidities), had Tyr concentrations of 666 µmol/L at T2. Conversely, more BC than CF infants had Lys and Thr concentrations below the 5th percentile (*p* < 0.05).

### Changes in plasma AA concentrations and associations with weight, length and HC growth

Patient flow charts and clinical characteristics across the two fortification groups, including only infants with plasma samples available at both T0, T1 and T2, are presented in Supplementary Fig. 1 and Supplementary Table S2, respectively. Combining data from the two fortification groups, the plasma concentrations of several (semi)EAAs (Arg, Ile, Leu, Lys, Met, Thr) and the combined values for BCAAs and EAAs increased from T0 to T2 (Supplementary Table S3).

HC growth from the start of fortification to 35 weeks PMA were positively associated with T0-T2 changes in 12 AAs (Ala, Arg, Asn, Cys, Gln, His, Ile, Lys, Met, Thr, Trp, Tyr), resulting in positive associations with T0-T2 changes for BCAAs, EAAs and total AAs (Table 3). The changes in AA concentrations were not associated with weight or length growth (Table 3). Associations between weight, length and head growth from the start of fortification to PMA 35 weeks and absolute AA concentrations (rather than changes in AA concentrations), are shown in Supplementary Table S4. The absolute concentrations of 14 AAs (Ala, Arg, Asn, Cys, Gln, Ile, Leu, Lys, Met, Pro, Thr, Trp, Tyr, Val) at T2 associated positively with changes in HC. This resulted in positive associations between HC growth and absolute BCAA, EAA and total AA concentrations at T2. No associations were found between HC growth and absolute AA concentrations at T0 and T1. Changes in body weight associated positively with the absolute concentrations of four (Arg, Ile, Lys, Trp), two (Cys, Lys) and eight AAs (Ala, Arg, Asn, Lys, Met, Thr, Trp, Tyr) at T0, T1 and T2, respectively. No associations were found between changes in length and absolute AAs concentrations at any time point (Supplementary Table S4).

**Table 3:** Associations between plasma amino acid (AA) changes from the start of fortification until two weeks of fortification and changes in Z-scores for weight, length and head circumference (HC) from the start of fortification until 35 weeks postmenstrual age

AA	Weight Coefficient (95% CI)·10^3^	*p*	Length Coefficient (95% CI)·10^3^	*p*	HC Coefficient (95% CI)·10^3^	*p*
Ala	0.600 (−0.394;1.595)	0.24	0.760 (−1.013;2.532)	0.40	1.309 (0.229;2.388)	0.02
Arg	0.256 (−2.114;2.627)	0.83	0.812 (−3.390;5.013)	0.70	3.490 (0.943;6.037)	<0.01
Asn	2.413 (−1.908;6.734)	0.27	4.678 (−2.952;12.307)	0.23	6.555 (1.934;11.176)	<0.01
Asp	−0.021 (−6.328;6.285)	0.99	−5.143 (−16.265;5.978)	0.36	0.560 (−6.275;7.395)	0.87
Cys	−0.230 (−3.082;2.621)	0.87	0.676 (−4.421;5.772)	0.79	3.753 (0.655;6.850)	0.02
Gln	0.285 (−0.160;0.729)	0.21	0.694 (−0.099;1.487)	0.09	0.609 (0.125;1.093)	0.01
Glu	−0.729 (−2.659;1.201)	0.46	−2.628 (-6.008;0.751)	0.13	−1.627 (−3.706;0.453)	0.12
Gly	−0.508 (−1.469;0.454)	0.30	0.189 (−1.517;1.896)	0.83	0.104 (−0.953;1.162)	0.85
His	0.647 (−2.785;4.080)	0.71	1.500 (−4.614;7.614)	0.63	4.087 (0.374;7.800)	0.03
Ile	0.115 (−3.031;3.261)	0.94	−0.306 (−5.958;5.347)	0.92	3.737 (0.287;7.187)	0.03
Leu	−0.623 (−2.422;1.177)	0.50	1.135 (−2.132;4.401)	0.49	2.008 (0.008;4.007)	0.05
Lys	0.589 (−0.628;1.806)	0.34	0.482 (−1.701;2.665)	0.66	2.200 (0.897;3.502)	<0.01
Met	3.410 (−5.433;12.253)	0.45	4.354 (−11.653;20.360)	0.59	16.297 (6.739;25.854)	<0.01
Phe	−2.524 (−7.432;2.384)	0.31	−0.700 (−9.584;8.185)	0.88	5.158 (−0.226;10.541)	0.06
Pro	−0.571 (−1.746;0.604)	0.34	0.558 (−1.544;2.660)	0.60	0.669 (−0.625;1.962)	0.31
Ser	−0.006 (−1.072;1.060)	0.99	0.847 (−1.041;2.734)	0.38	0.385 (−0.781;1.551)	0.52
Thr	0.221 (−0.517;0.960)	0.55	0.571 (−0.739;1.882)	0.39	0.801 (0.001;1.601)	0.04
Trp	2.441 (−3.989;8.871)	0.45	3.799 (−7.571;15.169)	0.51	9.196 (2.295;16.098)	<0.01
Tyr	0.746 (−0.461;1.953)	0.22	2.050 (−0.066;4.166)	0.06	1.511 (0.205;2.818)	0.02
Val	−0.542 (−2.421;1.338)	0.57	2.283 (−1.065;5.630)	0.18	1.635 (-0.423;3.693)	0.12
BCAA	−0.204 (-0.986;0.578)	0.61	0.595 (−0.815;2.004)	0.41	0.897 (0.035;1.760)	0.04
EAA	0.139 (−0.264;0.542)	0.50	0.264 (−0.455;0.983)	0.47	0.640 (0.207;1.073)	<0.01
Total	0.023 (−0.083;0.130)	0.67	0.104 (−0.085;0.293)	0.28	0.145 (0.030;0.259)	0.01

Analyses include only infants with plasma samples available at the start (T0), after one (T1) and two (T2) weeks of fortification across fortification groups (*n* = 182). General linear models were used and adjusted for intervention, gestational age at birth (above or below 29 weeks of gestation), small for gestational age and postnatal age at two weeks of fortification (T2). *AA* Amino acid, *BCAA* Branched-chain AA, *EAA* Essential AA, *HC* head circumference. Values are β-coefficients with 95% confidence intervals multiplied with 10^3^.

### Associations between AA and IGF-1 concentrations in plasma

Median plasma concentrations of IGF-1 were 24.1 (*n* = 172), 27.7 (*n* = 146) and 33.1 (*n* = 180) ng/ml at T0, T1 and T2, respectively. The increase in plasma IGF-1 concentrations from T0 to T2 was 6.8 ng/ml (*n* = 170) and positively associated with T0-T2 changes in Arg, Asp, Glu, Ser and Thr concentrations and EAA concentrations (Supplementary Table S5). The increases in IGF-1 concentrations were positively associated with changes in weight (β-coefficient·10^3^ ± SEM: 7.53 ± 2.83, *p* = 0.01) and HC (β-coefficient·10^3^ ± SEM: 9.47 ± 3.08, *p* < 0.01) from start of fortification to 35 weeks PMA.

### Associations between changes in AA concentrations and GA, weight at birth, and morbidities

The most immature infants (GA at birth<29 weeks) had lower plasma T0-T2 responses for 13 AAs (Arg, Asp, Cys, Gln, Glu, Gly, Ile, Leu, Lys, Met, Ser, Thr, Val, all *p* < 0.05), and to a large degree also SGA infants (Ala, Arg, Asp, Gly, Lys, Ser, Thr, Trp, all *p* < 0.05, Table 4). Infants diagnosed with BPD had lower plasma Arg and Gly responses after the start of fortification, while infants with ROP had lower plasma Arg and Gln responses, than infants without the diagnoses. Finally, sepsis diagnosis after the start of fortification was associated with reduced AA responses to fortification for Ala, Asn, Met, Trp and Tyr. The differences in AA responses could not be explained by differences in intake levels, as we found no differences in enteral volume or protein intakes within each of the five subgroups of infants shown in Table 4 (data not shown).

**Table 4:** Differences in plasma amino acid (AA) changes from start of fortification until two weeks of fortification in infant subgroups of low gestational age (GA) at birth, small for gestational age (SGA), bronchopulmonary dysplasia (BPD), retinopathy of prematurity (ROP) and late-onset sepsis (LOS).

AA	Present	GA < 29 weeks n = 101/182	*P*	SGA n = 39/182	*p*	BPD n = 35/182	*p*	ROP n = 16/182	*p*	LOS n = 21/182	*p*
Ala	Yes	22.4 ± 10.6	0.13	−1.7 ± 16.2	0.02	−3.9 ± 20.4	0.06	−10.9 ± 32.4	0.17	−16.5 ± 23.9	0.04
	No	45.4 ± 9.6		42 ± 8.0		41.3 ± 7.5		36.8 ± 7.3		39.0 ± 7.5	
Arg	Yes	26.4 ± 4.3	0.01	13.5 ± 6.4	<0.01	11.8 ± 5.9	0.01	0.5 ± 10.2	<0.01	15.6 ± 5.4	0.12
	No	42.7 ± 4.7		39.2 ± 3.6		38.9 ± 3.7		36.9 ± 3.3		36.0 ± 3.6	
Asn	Yes	12.6 ± 2.3	0.63	7.4 ± 3.5	0.15	10.5 ± 3.6	0.59	3.2 ± 5.8	0.08	2.5 ± 4.6	0.03
	No	11.2 ± 2.4		13.2 ± 1.8		12.3 ± 1.8		12.8 ± 1.7		13.2 ± 1.7	
Asp	Yes	−5.7 ± 1.7	0.01	−8.9 ± 2.9	<0.01	−6.7 ± 3	0.72	−1.1 ± 1.8	0.10	−5.0 ± 3.6	0.76
	No	0.2 ± 1.4		−1.5 ± 1.2		−2.2 ± 1.2		−3.3 ± 1.3		−2.8 ± 1.2	
Cys	Yes	−21.6 ± 3.4	0.04	−23.8 ± 5.9	0.18	−25.1 ± 6.5	0.43	−24 ± 10.1	0.74	−31.4 ± 9.6	0.09
	No	-11 ± 3.7		−15 ± 2.8		−14.9 ± 2.7		−16.2 ± 2.6		−15.0 ± 2.5	
Gln	Yes	−86.3 ± 22.1	0.04	−118.3 ± 31.8	0.05	−99.3 ± 35.5	0.69	−207.6 ± 62.7	0.02	−137.3 ± 62.5	0.19
	No	−17 ± 23.1		−38.3 ± 18.5		−45 ± 18.1		−40.8 ± 16.3		−44.8 ± 16.3	
Glu	Yes	−12.9 ± 6.2	0.01	−22.8 ± 15.5	0.14	−26.6 ± 15	0.34	−11.7 ± 4.5	0.65	−15.5 ± 7.1	0.18
	No	4.5 ± 3.1		−0.4 ± 2.2		−0.1 ± 2.9		−4.5 ± 4.1		−3.8 ± 4.2	
Gly	Yes	−33.5 ± 11.4	<0.01	−58.3 ± 19.7	<0.01	−76.1 ± 23.1	<0.01	−63.4 ± 28.2	0.26	−40.2 ± 20.7	0.65
	No	13.9 ± 10		0.1 ± 8.2		2.8 ± 7.6		−7.5 ± 8.1		−8.8 ± 8.5	
His	Yes	-7.4 ± 3.2	0.10	-9.9 ± 4.3	0.19	−9.7 ± 5.2	0.51	−8.8 ± 7.7	0.72	−14.0 ± 6.8	0.13
	No	−0.3 ± 2.5		−2.7 ± 2.4		−2.9 ± 2.3		−3.8 ± 2.2		−2.9 ± 2.2	
Ile	Yes	14 ± 2.4	0.01	17 ± 4.8	0.65	12.8 ± 4.4	0.72	7.6 ± 6.4	0.36	5.1 ± 3.7	0.10
	No	26 ± 4.1		20 ± 2.6		20.9 ± 2.6		20.5 ± 2.4		21.2 ± 2.5	
Leu	Yes	1.9 ± 4.6	<0.01	2.2 ± 8.8	0.14	−0.9 ± 8.5	0.69	−11.1 ± 12.8	0.35	−15.2 ± 9.1	0.08
	No	29.9 ± 6.8		17.7 ± 4.6		18 ± 4.6		16.8 ± 4.3		18.2 ± 4.4	
Lys	Yes	46.9 ± 7	0.01	32.6 ± 15.6	0.02	39.9 ± 13	0.63	16.4 ± 19.3	0.19	22.8 ± 15.7	0.17
	No	77.6 ± 10		68.2 ± 6.2		65.5 ± 6.7		64.8 ± 6.2		65.5 ± 6.4	
Met	Yes	4.9 ± 1.1	<0.01	6.7 ± 1.8	0.82	4.9 ± 1.6	0.83	1.4 ± 3.2	0.12	1.4 ± 2.4	0.04
	No	10.2 ± 1.2		7.4 ± 0.9		7.8 ± 0.9		7.8 ± 0.8		8.0 ± 0.9	
Phe	Yes	−8.4 ± 2.0	0.09	−8.7 ± 3.2	0.43	−9.6 ± 3.3	0.56	−13.2 ± 4.7	0.18	−12.0 ± 3.5	0.17
	No	−3.3 ± 2.1		−5.5 ± 1.7		−5.3 ± 1.7		−5.5 ± 1.6		−5.4 ± 1.6	
Pro	Yes	−36.2 ± 8.7	0.07	−40.3 ± 11.7	0.26	−48 ± 17.1	0.26	−58 ± 25	0.18	−48.6 ± 20.0	0.26
	No	−12.7 ± 8.9		−21.8 ± 7.3		−20.5 ± 6.6		−22.7 ± 6.4		−22.8 ± 6.6	
Ser	Yes	−29.6 ± 10.5	0.02	−50.5 ± 14.6	<0.01	−53.4 ± 16.7	0.07	−48.9 ± 18.6	0.42	−50.2 ± 17.2	0.20
	No	4.3 ± 8.5		−4.7 ± 7.8		−5.3 ± 7.6		−11.2 ± 7.5		−9.9 ± 7.6	
Thr	Yes	−13.4 ± 14	<0.01	−33.3 ± 27	<0.01	−43.7 ± 25.2	0.11	−63.8 ± 33.5	0.16	−42.1 ± 31.8	0.26
	No	58.3 ± 13.6		32.6 ± 10.4		33.3 ± 10.7		26.4 ± 10.5		26.4 ± 10.6	
Trp	Yes	11.1 ± 1.4	0.66	6.4 ± 3	0.01	11.5 ± 2.3	0.68	6.6 ± 3.5	0.35	4.0 ± 2.5	0.03
	No	12.3 ± 1.8		13.1 ± 1.1		11.7 ± 1.3		12.1 ± 1.2		12.6 ± 1.2	
Tyr	Yes	24.2 ± 7.4	0.09	25 ± 10.4	0.37	24.6 ± 9.1	0.90	40 ± 36.2	0.69	−3.6 ± 10.5	0.03
	No	3.7 ± 10.1		12.3 ± 7.3		12.8 ± 7.3		12.7 ± 5.8		17.5 ± 6.8	
Val	Yes	2.7 ± 5.8	0.01	2.4 ± 7.9	0.28	−0.6 ± 9	0.45	−8.4 ± 14.2	0.14	−9.1 ± 10.7	0.05
	No	23.2 ± 6.1		14.4 ± 4.9		14.8 ± 4.8		13.8 ± 4.4		14.5 ± 4.6	

Analyses include only infants with plasma samples available at the start (T0), after one (T1) and two (T2) weeks of fortification across fortification groups. General linear models were used and adjusted for intervention, gestational age (above or below 29 weeks of gestation) at birth, small for gestational age and postnatal age at two weeks of fortification (T2) when these were not the variables of interest. *AA* amino acid, *BPD* bronchopulmonary dysplasia, *GA* gestational age, *LOS* late-onset sepsis, *ROP* retinopathy of prematurity, *SGA* small for gestational age. Values are means ± SEM in µmol/L.

### Fortification-related changes in plasma proteins as assessed by proteomics

A total of 701 plasma proteins were identified and annotated after statistical filtration, while 197 and 186 protein groups were identified as the “effect protein groups” on T1 and T2, respectively. No proteins groups were identified as “exposure protein groups” for BC fortification. Unsupervised principal component analysis (PCA) was performed using protein abundance data from the plasma proteome with Permutation-based FDR *p*-value correction and imputation of missing values. No clear separation was found between the fortification groups at each time point (Supplementary Fig. 2). At T1 and T2, 10 and 3 proteins showed *p* < 0.05 between groups, but none of these remained significant after FDR adjustment (*n* = 87–95, Supplementary Table S6). Given that negligible treatment effects were observed on individual plasma proteins by the proteomics analyses, further analyses were focused only on the overall plasma proteome changes between sampling time points across fortification groups. We identified 211 effect protein groups but none of these showed any differences in expression in plasma across T0, T1 or T2 (Supplementary Table S7).

## Discussion

In the current study, we show that BC fortification of human milk increased the plasma concentrations of 12 AAs relative to CF in VPIs. Analysed across fortification groups, as an explorative part of the study, HC growth was significantly associated with increments in, and absolute concentrations of, specific AAs, especially EAAs. Yet, the effect sizes were small, the concentrations of circulating AAs were highly variable and for many AAs, their plasma responses to fortification were negatively affected by birth status (e.g., GA, SGA) and morbidities, like LOS. Thus, circulating AA concentrations are unlikely to be good predictors of protein intake, growth or morbidities for individual VPIs.

Considering our results, attention to possible AA imbalances is required for the new BC fortifier. Despite the observed elevated AA concentrations, growth rates were unaffected by BC fortification, as reported in our previous studies.^23,34^ This may partly be explained by reduced Lys content in the BC versus CF product, causing lower Lys concentrations in plasma (-16% at T1 and -10% at T2, Fig. 2, *p* < 0.05) to be limiting for protein accretion. Similarly, when referenced against values reported for preterm infants with similar age and diets,^16^ more BC infants had Lys concentrations below the chosen reference range (*p* = 0.02, Supplementary Table S1). Lys requirements are high in the first month of life, as demonstrated in term, formula-fed infants,^35^ and absolute Lys concentrations at both T0, T1 and T2 were positively associated with body weight growth in the current study. Conversely, lower Met, Leu and Ile contents in the BC product were not associated with reduced concentrations of these AAs in plasma, probably because these EAAs were already provided in excess of requirements in both groups. Additionally, Thr approached reduced plasma concentrations in BC versus CF infants (*p* = 0.13 at T2, Fig. 2) and more BC infants had Thr concentrations below the chosen reference range (*p* < 0.01, Supplementary Table S1). Thr is central to glycoprotein production in the intestinal mucin layer, protecting the immature intestine against bacterial translocation.^36,37^ Hence, specific addition of Lys and Thr should be considered for use of BC as a fortifier for VPIs.

Preterm infants may have an immature ability to metabolize AAs and excess supply could potentially lead to toxicity, although the toxicity threshold for total or individual AAs is unknown.^38,39^ BC fortification increased the plasma concentrations of Tyr and Phe at T1 and T2 (*p* < 0.001, Fig. 2), and more BC than CF infants had Tyr concentrations above the chosen reference range (*p* < 0.01, Supplementary table S1). Phe is metabolized to Tyr and concerns about hypertyrosinemia-induced neurotoxicity have been raised.^40–42^ One infant in the BC group had Tyr concentrations above the threshold in patients with genetic hypertyrosinemia (>500 μmol/L). However, sparse evidence prohibits any firm conclusions regarding recommendations for upper Tyr levels during transient diet-induced hypertyrosinemia^38,39^ and the clinical implications of BC-induced elevated Tyr concentrations are uncertain. Generally, slight AA imbalances after BC-fortification did not lead to excessive AA oxidation in this study, as indicated by similar BUN values between groups, as reported previously.^23^

The main differences in protein quality between BC and CF products are the presence of casein and immunoglobulins in BC and the pre-hydrolysis of bovine whey proteins in CF.^23^ In preterm pigs, BC feeding induced clear gut protection in the first 1–2 weeks of life relative to CF feeding,^21,43,44^ at least in part via intact milk bioactive proteins, such as immunoglobulins, lactoferrin and various growth factors.^17,24,29,45^ Potentially, casein and immunoglobulins are digested more slowly than hydrolysed whey protein, contributing to altered bioavailability of selected AAs.^46–48^ Casein contains more Arg, Phe and Tyr, and less Ala, Leu, Lys and Thr, than whey protein.^49^ This may partly explain the observed increased concentrations of Arg, Phe and Tyr, and the Lys and Thr deficiency in BC infants (see above). In contrast, our study did not indicate any BC-specific deficiency in Leu supply, a well-recognized EAA and BCAA stimulating growth in infants via the mTOR pathway and local IGF-1 release,^9^ and Ala concentrations were generally increased in BC infants. The BC infants had a slightly higher intake of human milk and received 0.2–0.3 g/kg/d more protein than the CF group at T1-T2, to attain similar growth rates between groups (Table 2). Adjustment for enteral milk/protein intake, or baseline values, had little impact on the overall results regarding the BC fortification effects, but the BC effects on Ala (and Asn) concentrations at T2 disappeared after additional adjustments for enteral volume intake.

In contrast to our preterm pig studies,^20,21,43,50–52^ BC fortification did not provide marked clinical benefits in hospitalized VPIs,^23^ although it may improve bowel habits.^53^ Importantly, the provided BC amounts were much higher in the pig studies, exerting clear gut and immune protection in the first week of life,^20,43,50^, but fewer benefits in the second week.^21,51^ By randomization, relatively more infants were allocated to the BC group in Eastern Denmark, potentially contributing to a lower breastfeeding rate in BC-fortified infants and impacting AA levels and clinical outcomes.^23^ However, with >80% of the included infants receiving primarily MOM, it was not meaningful to study the effects of milk type (MOM, DHM, mixed) in this study.

Analysed across fortification groups, we show that the plasma concentrations of important EAAs (Ile, Leu, Lys, Met, Thr) increased in response to the start of human milk protein fortification and that HC, but not weight or length, growth were positively associated with increments in 12 specific AAs and total AAs from T0 to T2, including increases in most EAAs (Table 3). Also, the fortification-related responses of Leu and Ile were positively associated with HC increments (*p* = 0.05 and 0.03, respectively, Table 3), leading to an overall positive association between BCAA and HC growth. These findings are important as HC growth has been linked with brain and cognition development in preterm infants.^54–56^ On the other hand, the effect sizes of individual AA-HC associations were small, hence other factors than AAs play a role in HC growth, and excess AAs may not further improve growth, but mainly serve as a substrate for oxidation and energy supply. In some studies, increased enteral and parenteral AA supply induced HC growth,^14,57^, while other studies showed no changes, or even reduced HC.^58–60^ Endocrine or paracrine regulators of protein synthesis across organs (e.g., brain, gut, liver, muscle, bone) may play a role in postnatal growth. One of the most well-known regulators is IGF-1^61^ which was also associated with growth across the VPIs in our study. The results suggest that specific AAs (Arg, Asn, Glu, Ser, Thr) may mediate fortification-induced increases in plasma IGF-1. Interestingly, we did not observe any associations between changes in IGF-1 and Leu concentrations, despite that Leu mediates muscle growth partly via IGF-1 effects.^8,62,63^

While dietary protein clearly affected circulating AAs, differential protein intake from the two fortifiers did not affect overall plasma protein composition, as assessed by proteomics. No changes in the overall plasma protein pattern, nor in the concentrations of individual proteins, were seen across T0, T1 and T2, supporting that protein composition in plasma changes little after the first week of life in preterm infants.^26^ Overall, our plasma proteomics data indicate that the composition of circulating plasma proteins is relatively stable and not affected by the source and level of dietary protein given to milk-fed VPIs.

In our study, infants born at low GA and SGA had reduced fortification-induced increments of several AAs, both non-essential and essential (e.g., Arg, Asp, Gln, Glu, Gly, Leu, Lys, Ser, Thr). Since their enteral volume and protein intakes were similar to remaining infants, these subgroups may need adjusted AA fortification, possibly due to their impaired dietary uptake (e.g., immature digestion or absorption), higher AA catabolism (e.g., systemic inflammation) or altered AA utilization. Further studies are required to show if a targeted supply of specific EAAs improves growth parameters for these subgroups of preterm infants.

This study has several limitations. We could not tightly control the pre-prandial timing of blood sampling from infants, which could induce further variation in measured AA concentrations. For the explorative part of the study, we only included infants with AA measurements available at all three sampling time points. Although the total sample size remained high (*n* = 182), this may have induced sampling bias. Also, a slight difference in age at the start of fortification could have impacted the analysis (e.g., median was one day earlier in the BC vs. CF group^23^). This may be explained by the clinical personnel being positively biased towards the novel fortifier, as the fortification products could not be blinded.

No universal reference exists for optimal plasma AA concentrations for preterm infants. We chose a clinical reference cohort with GA at birth, postnatal age, protein fortifier (FM85 from Nestlé) and weight gain similar to that in our study cohort.^16^ Thus, we believe that our AA values were referenced against near-optimal conditions in a comparable group of preterm infants. Finally, our explorative statistical approach prevents us from drawing firm cause-effect conclusions. Further studies are therefore required to fully understand the effects of dietary protein type and level on circulating AAs and specific plasma proteins.

In conclusion, fortification per se and the type of fortifier affect circulating AA concentrations in the first weeks after start of fortification in VPIs. The use of BC as an alternative, bioactive fortifier may benefit from adjustment of AA intakes by adding synthetic AAs (e.g., Lys, Thr). Yet, it remains unclear if BC fortification induces any clinical benefits for VPIs when used beyond the first week of life and in relatively small amounts. At the population level, the AA increases in plasma after the start of fortification, associated positively with HC growth, but, interestingly, not with body weight and length growth. Yet, the observed effect sizes were small, and the AA responses to fortification were affected by baseline clinical variables such as GA and weight at birth. Many factors beyond enteral and parenteral protein intake affect the complex interplay among growth, clinical variables and circulating AA concentrations. This complexity limits the practical application of individual AAs, or groups of AAs, as biomarkers of protein intake quality, body growth or other clinical outcomes for individual VPIs.

## Supplementary information

Supplementary figures

Supplementary table S6

Supplementary table S7

Supplementary table S1

Supplementary table S2

Supplementary table S3

Supplementary table S4

Supplementary table S5

## Data Availability

Data described in the manuscript, code book and analytic code will be made available upon request pending to corresponding author. All clinical data were entered into the online database, REDCap, in OPEN, in the Region of Southern Denmark. The MS proteomic data were deposited to the ProteomeXchange Consortium via the PRIDE partner repository with the dataset identifier PXD053348.
