# Supplementary material for: Plasma amino acids after human milk fortification and associations with growth in preterm infants
Source: Pediatr Res. 2025 May 18;99(7):2614–23. doi: 10.1038/s41390-025-04126-6 (PMC13485694; doi:10.1038/s41390-025-04126-6)
Supplement: Supplementary file 1 [file 41390_2025_4126_MOESM1_ESM.pdf]

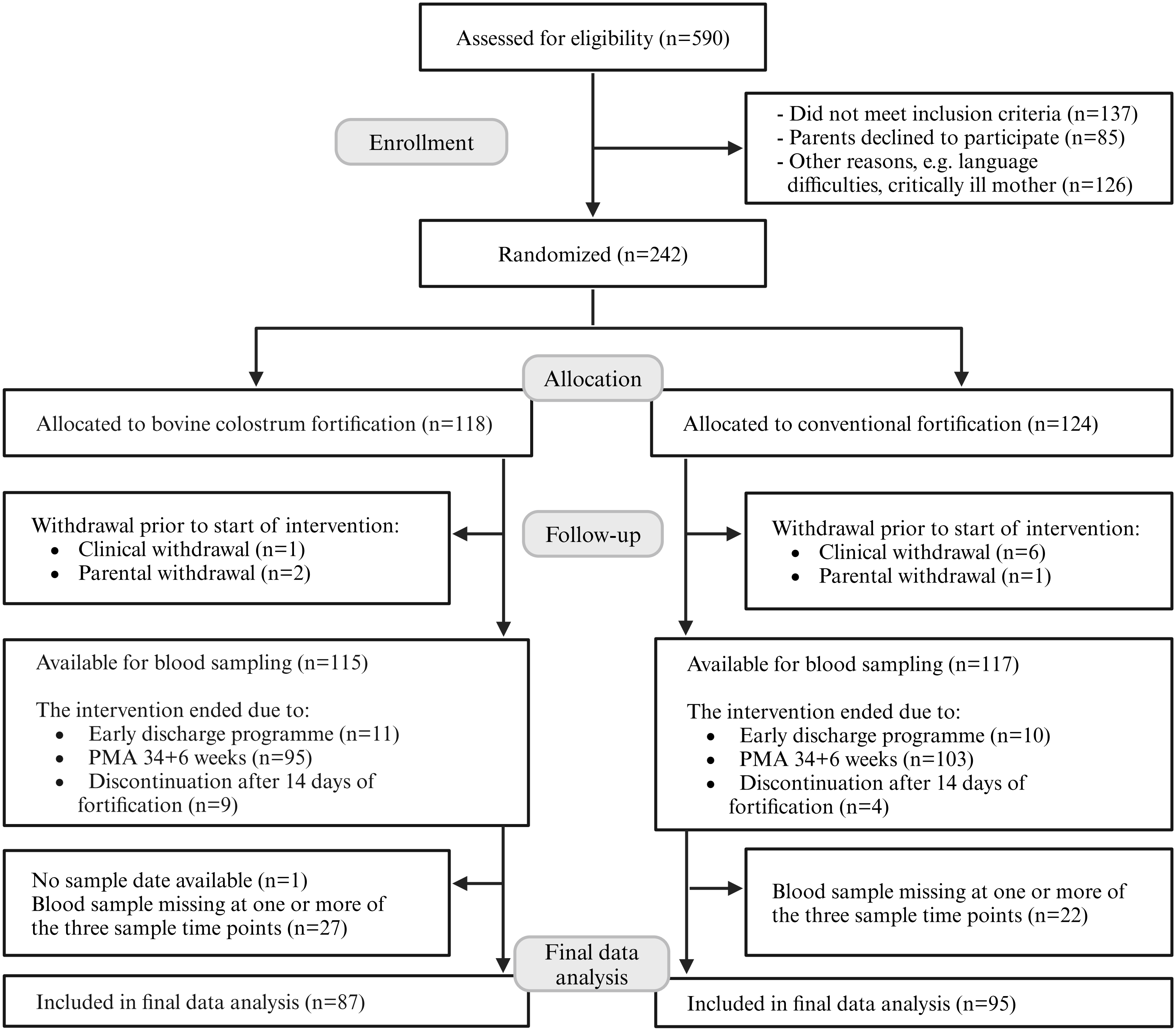

Supplementary figure 1. Participant flow chart. Created with BioRender.com

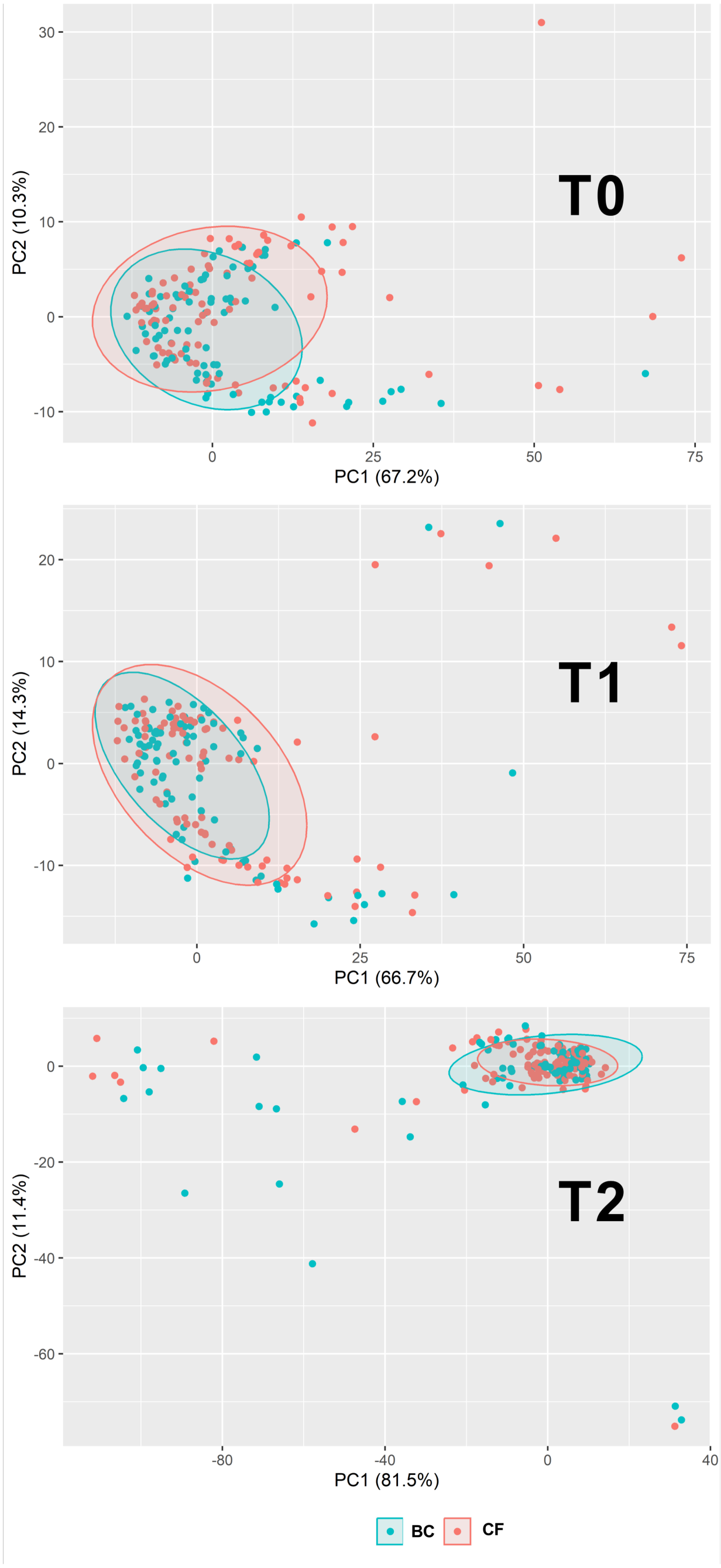

Supplementary figure 2. Principle component analysis (PCA) illustrating the proteome profile between BC and CF infants at the start (T0), after one (T1) and two (T2) weeks of fortification. The PCA analysis was based on all annotated plasma proteins at each sampling time point by liquid chromatography mass spectrometry based proteomics. Scores of first two principal components from PCA are plotted. Blue: BC, Red: CF.
